# Vertically inherited microbiota and environment‐modifying behaviors indirectly shape the exaggeration of secondary sexual traits in the gazelle dung beetle

**DOI:** 10.1002/ece3.10666

**Published:** 2023-10-31

**Authors:** Patrick T. Rohner, Armin P. Moczek

**Affiliations:** ^1^ Department of Biology Indiana University Bloomington Bloomington Indiana USA; ^2^ Department of Ecology, Behavior and Evolution University of California, San Diego La Jolla California USA

**Keywords:** allometry, host–microbiome interaction, nutritional plasticity, organism–environment interactions, sexual dimorphism

## Abstract

Many organisms actively manipulate the environment in ways that feed back on their own development, a process referred to as developmental niche construction. Yet, the role that constructed biotic and abiotic environments play in shaping phenotypic variation and its evolution is insufficiently understood. Here, we assess whether environmental modifications made by developing dung beetles impact the environment‐sensitive expression of secondary sexual traits. Gazelle dung beetles both physically modify their ontogenetic environment and structure their biotic interactions through the vertical inheritance of microbial symbionts. By experimentally eliminating (i) physical environmental modifications and (ii) the vertical inheritance of microbes, we assess the degree to which (sym)biotic and physical environmental modifications shape the exaggeration of several traits varying in their degree and direction of sexual dimorphism. We expected the experimental reduction of a larva's ability to shape its environment to affect trait size and scaling, especially for traits that are sexually dimorphic and environmentally plastic. We find that compromised developmental niche construction indeed shapes sexual dimorphism in overall body size and the absolute sizes of male‐limited exaggerated head horns, the strongly sexually dimorphic fore tibia length and width, as well as the weakly dimorphic elytron length and width. This suggests that environmental modifications affect sex‐specific phenotypic variation in functional traits. However, most of these effects can be attributed to nutrition‐dependent plasticity in size and non‐isometric trait scaling rather than body‐size‐independent effects on the developmental regulation of trait size. Our findings suggest that the reciprocal relationship between developing organisms, their symbionts, and their environment can have considerable impacts on sexual dimorphism and functional morphology.

## Graphical abstract

Many organisms actively manipulate the environment in ways that have the potential to feed back on their own development, a process referred to as developmental niche construction. We assess whether environmental modifications made by developing dung beetles impact the environment‐sensitive expression of secondary sexual traits. Our findings suggest that the reciprocal relationship between developing organisms, their symbionts, and their environment can have considerable, although indirect, impacts on sexual dimorphism and functional morphology.

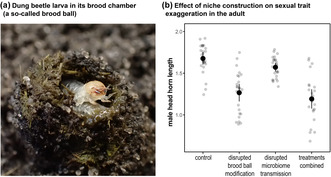

## 1 INTRODUCTION

The environmental variation that organisms experience during development has major impacts on their phenotypes. Rooted in the intrinsic context‐dependency of development, such environmental plasticity is ubiquitous and has long been recognized as a major factor in ecology and evolution (Pfennig, 2021; West‐Eberhard, 2003). However, the role of the organism and its potentially reciprocal interactions with its environment in shaping this dynamic is still poorly understood (Sultan, 2015). For example, many organisms possess the capacity to actively manipulate the environment they themselves experience—and subsequently respond to—during ontogeny. These feedbacks can arise, for instance, via physical environmental manipulations or by biasing the biotic communities organisms encounter (Gilbert et al., 2015). Such “developmental niche construction” (Stotz, 2017; Uller & Helantera, 2019) alters ontogenetic environments and has the potential to feed back to the organism's phenotype if its development is sensitive to the constructed environment (Clark et al., 2020; Donohue, 2005; Odling‐Smee et al., 2013). Developmental niche construction may thus drive phenotypic variation in traits that are especially sensitive to environmental conditions, such as secondary sexual traits. Yet, few studies have been able to examine these conjectures experimentally. Using an experimental manipulation of organisms' ability to shape their physical and (sym)biotic environment, we assess the degree to which the expression of secondary sexual traits depends on the interactions between developing organisms, their symbionts, and their environment.

Sexually dimorphic (or sex‐limited) morphological structures, such as horns, antlers, or ornamental feathers, are often costly in terms of survival and the energetic expenditure of growing and maintaining them (Grafen, 1990; O'Brien et al., 2019; Rowe & Houle, 1996). Because environmental quality is a major determinant of the relative (or marginal) costs of producing a sexual signal, secondary sexual trait exaggeration is often environmentally plastic (i.e., dependent on the “phenotypic quality” Zahavi, 1977). For instance, the expression of morphological traits functioning as ornaments or weapons in intra‐ and intersexual competition are often limited to males and tied to the bearer's nutritional condition or social status (Cotton et al., 2004; Emlen et al., 2012; Pryke & Andersson, 2005; Rohner & Blanckenhorn, 2018; Ruell et al., 2013). This causes large males to frequently develop disproportionately larger secondary sexual traits (hyperallometry), and the degree to which trait size scales with body size may itself be dependent on environmental quality (“allometric plasticity” Emlen, 1997b; Rhebergen et al., 2022). If the development of secondary sexual traits is more environment‐sensitive than that of others, their relative size may also be particularly dependent on the way or extent to which organisms actively shape their own ontogenetic environment. Specifically, if (adaptive) environmental modifications increase environmental quality and thereby shape the marginal costs of producing a sexual trait, it may afford otherwise low‐quality individuals to produce a disproportionately large sexual signal. If so, the interactions between organisms and their ontogenetic environments would constitute a major determinant of variation in secondary sexual trait expression. However, the degree to which secondary sexual traits and their scaling are affected by these interactions remains unclear, let alone whether they are more affected than other types of traits. We here start to explore this relationship in dung beetles.

Onthophagine dung beetles have received considerable attention due to their diversified, condition‐dependent, and often greatly exaggerated secondary sexual traits, such as forelegs and head and thoracic horns used in male combat or courtship (Kotiaho, 2002; Moczek & Emlen, 2000; Rohner et al., 2021, 2023). For horns in particular, the roles of environmental conditions—specifically quantity and quality of larval nutrition—in the determination of size and degree of exaggeration are well understood (Emlen, 1997c; Moczek, 1998). More recently, onthophagine beetles have also attracted further attention following the discovery of elaborate environment‐modifying behaviors (Schwab et al., 2017): larvae of various species develop in individual underground brood chambers (so‐called “brood balls”) constructed by the mother (Hanski & Cambefort, 1991). Upon hatching, larvae physically modify their brood ball by continuously feeding on its content, excreting back into their brood ball, and re‐eating the increasingly modified material (Estes et al., 2013; Schwab et al., 2016). Preventing larvae from manipulating their brood ball leads to smaller adult size, extended development time, and reduced reproductive output as adult (Schwab et al., 2016), suggesting that the environmental modifications made by the larva enhance environmental quality. In addition to these physical modifications, dung beetles also shape their biotic environment. During oviposition, mothers place each egg onto a small amount of their own excrement, the so‐called “pedestal,” representing a microbial inoculate that is consumed by the larva upon hatching. In so doing, the mother's gut microbiome is transmitted vertically to her offspring (Estes et al., 2013). As the developing larva continually defecates, works its own excrement into the brood ball, and then re‐eats the resulting composite, the maternally inherited gut microbiome is spread throughout the brood ball (Schwab et al., 2016, 2017). These vertically transmitted microbial communities have been shown to be host species and population‐specific (Parker et al., 2020) and to yield deleterious fitness consequences if withheld, including a reduction in adult size (Parker et al., 2019, 2021; Parker & Moczek, 2020; Schwab et al., 2016). Physical and (microbiome‐mediated) biotic environmental modifications thus seem to play major roles in determining the nutritional environment, the resulting growth potential of a larva, and the size at adult eclosion, which in turn corresponds to individual quality or condition (Bonduriansky, 2007a; Emlen et al., 2012; Rohner & Blanckenhorn, 2018). However, whether these dynamics generally affect secondary sexual trait exaggeration remains unclear.

**FIGURE 1:**
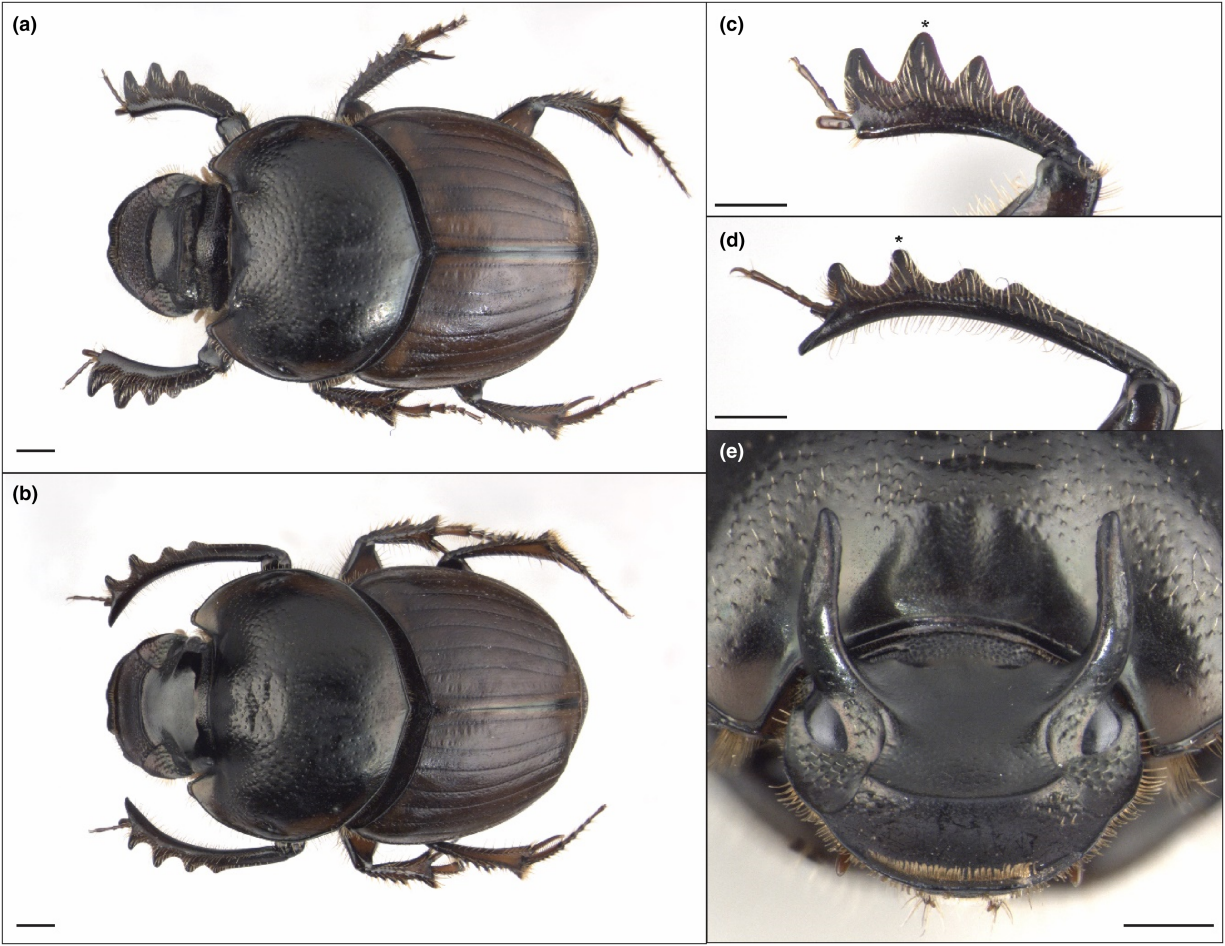
Sexually dimorphic morphology in *Digitonthophagus gazella*. Panels (a) and (b) show the overall morphology of females and males, respectively. While females have relatively stout, short, and more heavily toothed fore tibiae (c), male fore tibiae are strongly elongated, much narrower, and possess smaller teeth (d). Head horns (e) are used in male–male combat and are only expressed in males. Scale bar = 1 mm. The position of the second tibial tooth is indicated with an asterisk.

Because dung beetles both possess condition‐sensitive secondary sexual trait development and larvae modify the developmental environment to which they themselves respond, this raises the possibility that organism‐driven environmental modifications may also shape variation in secondary sexual trait expression. Here, we combine a common garden design with an experimental elimination of (sym)biotic and physical environmental modifications to test whether the presence of a maternally inherited microbiome and a larva's ability to physically manipulate its environment shape secondary sexual trait expression in the gazelle dung beetle *Digitonthophagus gazella* (Fabricius, 1787; Figure 1). We do so by focusing on several traits that vary in the degree and direction of sex‐specific exaggeration: (i) exaggerated male head horns, a strongly nutritionally plastic weapon only present in males (Casasa et al., 2020); (ii) the length of the fore tibia, which is exaggerated in males and used during mating (Rohner et al., 2021); (iii) the width of the fore tibia, which is exaggerated in females and used to construct underground tunnels (Linz et al., 2019; Macagno et al., 2016); and (iv) the length and width of the elytra (i.e., the modified first pair of wings), which shows comparatively minor differences in relative size and shape across the sexes (Figure 1). If environmental modifications play major roles in the development of secondary sexual trait expression, we expect individuals able to construct their ontogenetic environment to develop disproportionately large secondary sexual traits relative to their body size (an index of individual condition or quality). We predict further that this effect should be strongest for the most exaggerated and nutritionally plastic traits (i.e., the male head horns, followed by the length of the male fore tibia, and the width of the female tibia), but absent for traits with a minimal degree of sexual dimorphism and exaggeration (elytron length and width). We find that ontogenetic environmental modifications indeed shape secondary sexual trait expression and do so variably for different trait classes. Yet, we also show that most of this impact can be explained by allometry, that is, effects on overall body size coupled with trait‐specific non‐isometric scaling. However, we posit that regardless of the precise mechanisms, developmental niche construction‐mediated shifts in the absolute size of secondary sexual traits are nevertheless expected to have functional consequences in this and likely many other species.

## 2 MATERIALS AND METHODS

### 2.1 General laboratory rearing and experimental manipulation

*Digitonthophagus gazella* (Fabricius, 1787) were collected in March 2021 near Pretoria, South Africa, and sent to Indiana University, Bloomington, USA, where they were kept under standard laboratory conditions. To obtain laboratory‐reared F1 individuals, we repeatedly transferred 4–6 wild‐caught (F0) females from the laboratory colony into rectangular oviposition containers (27 × 17 × 28 cm) filled with a sterilized sand–soil mixture and topped off with ca. 800 g of defrosted cow dung. After 5 days, brood balls were sifted from the soil and kept in plastic containers filled with soil at a constant 29°C.

Newly emerged F1 offspring were kept in single‐sex containers at 26°C for at least 7 days. Thereafter, 30 half‐sib families consisting of three females (dams) and one male (sire) were housed in separate containers equipped with soil and defrosted cow dung for at least 4 days. Females were then transferred into individual oviposition containers (27 × 8 × 8 cm) filled with a sterilized sand–soil mixture and 200 g of defrosted cow dung (see Rohner & Moczek, 2020) and kept at 29°C. After 5 days, all the brood balls produced were collected and opened. F2 offspring were reared in standardized, artificial brood balls as described previously (Shafiei et al., 2001). In brief, we opened all natural brood balls and transferred eggs individually into the wells of standard 12‐well tissue culture plates. Each well was provisioned with 2.9 (±0.1) grams of defrosted, thoroughly homogenized cow dung. We only used dung from hay‐fed cows, which represents a more challenging diet compared to the dung from grass‐fed cows (Rohner & Moczek, 2021). Plates were kept at 29°C and checked every 24 h for hatching. All F2 were subjected to two fully factorial manipulations of a larva's ability to shape its biotic and physical ontogenetic environment:

#### 2.1.1 Microbiome manipulation

To manipulate the vertical transmission of microbial symbionts, we surface sterilized half of all eggs with 200 μL of a 1% bleach and 0.1% Triton‐X 100 solution, followed by two rinses with deionized water (see Macagno & Moczek, 2022; Parker et al., 2019; Schwab et al., 2016). Eggs in the control treatment were rinsed with deionized water only. Eggs were then placed in an artificial, standardized brood ball, either with (“intact microbiome transmission”) or without (“disrupted microbiome transmission”) the extracted maternal pedestal.

#### 2.1.2 Manipulation of larval environment‐modifying behavior

The capacity of larvae to manipulate their brood ball was experimentally hampered by relocating individuals into a new artificial brood ball 4, 7, 10, and 13 days after eggs were initially transferred using featherweight forceps (see Dury et al., 2020; Schwab et al., 2017). This procedure exposes the developing larvae repeatedly to new, unprocessed cow dung and prevents the accumulation of physical modifications applied to the brood ball (“disrupted brood ball modification”; note that Schwab et al., 2017 relocated larvae every 48 h throughout larval development, starting 24 h after hatching; given our sample size of 1228 individuals, this procedure was logistically unfeasible). The respective ages at which brood balls were exchanged were chosen to cover the developmental time where larvae grow most (Rohner & Moczek, 2021). Our approach also ensured that each individual was exposed to the same number of manipulations, irrespective of development time. In the control treatment, larvae were allowed to complete their development in their original well. To account for the potential stress induced by repeatedly relocating larvae into new wells, larvae were removed from their brood ball, held with featherweight forceps for approximately 3 s, and placed back in their original well 4, 7, 10, and 13 days after eggs were transferred into a new plate (“intact brood ball modification”).

Individuals were checked daily until their emergence as adults. Once the adult cuticle was fully hardened, individuals were sacrificed and stored in ethanol. We imaged the adult thorax, abdomen, and foreleg using a Scion camera mounted on a Leica MZ 16 stereomicroscope. We measured pronotum width as an estimate for body size (see Rohner, 2021), tibia length, as well as elytra length using the linear distance between defined landmarks using tpsDig2 (Rohlf, 2009; see Figure S1 for the location of landmarks used for measurements). As a measure of the width of the fore tibia, we measured the height of the second tibial tooth as indicated in Figure S1. This tooth is the largest of all four tibial teeth and is much longer and broader in females compared to males (see Figure 1). Male head horns were photographed using a Pixelink PL‐D797CU‐T camera mounted on a Leica MZ 16 stereomicroscope and measured as the length of the outline between the eye and the tip of the horn (Rohner et al., 2020; also see Figure S1). Occasionally, prepupae position themselves in such a way that the development of one of the horns is impeded during pupal development, leading to characteristic deformations and asymmetries in horn morphology. When the left and right horns of the same individual differed in length, we only measured the longer horn. If both horns were damaged or malformed, individuals were excluded from the analysis.

### 2.2 Statistical analysis

To test for effects on secondary sexual trait exaggeration in tibia and elytron length and width, we fitted log trait size as a function of log body size, the experimental treatments, sex, and all interactions using the R packages *lmerTest* (Kuznetsova et al., 2017) and *lme4* (Bates et al., 2015) in R version 4.2.2 (R Core Team, 2021). Sire, dam nested within sire, as well as the 12‐well plate individuals were reared in, were added as random effects. Non‐significant interactions were removed except for the interactions between the experimental treatments and sex, as these were of a priori interest. We calculated partial *R* ^2^ values as effect sizes for fixed effects. Partial *R* ^2^ for main effects was estimated using models excluding interaction effects. The variances explained by the interactions were estimated in separate models (see Stoffel et al., 2021). We only included individuals for which measurements for all traits (apart from male‐limited horns) were available (*n* = 903). To visualize variation in relative size, we extracted residual trait size from an ordinary linear regression of log trait size against log body size, combining both sexes and all treatments in all cases. Residuals were then averaged by sex and sire and plotted by treatment.

Next, we assessed whether our experimental manipulations affected horn length and its scaling with overall body size. Because horn length shows a non‐linear relationship with body size, we fitted and compared two separate four‐parameter log‐logistic models in the R package *drc* (Ritz et al., 2015). The first model included a single curve for all individuals (i.e., a common allometry for all treatments), while in the second model we fitted separate curves for all four treatment combinations. We chose the best‐fitting model based on Akaike's Information Criterion (AIC). In addition, we extracted residual horn length and fitted it as a function of our experimental treatments using sire, dam nested within sire, as well as plate as random effects. The total sample size for this male‐limited trait was *n* = 453.

## 3 RESULTS

### 3.1 Body size

**FIGURE 2:**
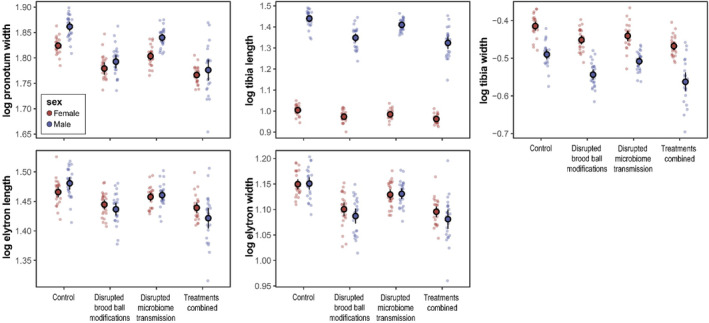
Effect of experimental treatments on mean absolute trait size and corresponding 95% confidence limits (*n* = 900 individuals with data available for all traits (offspring of 67 dams and 25 sires)). Points indicate treatment‐specific sire means.

In agreement with previous studies, we find that body size, as measured by the width of the pronotum, was reduced when preventing larvae from manipulating their brood ball (*χ* ^2^_(1)_ = 246.49, *p* = <.001, partial *R* ^2^ = .23) or when maternal microbes were withheld (*χ* ^2^_(1)_ = 61.64, *p* = <.001, partial *R* ^2^ = .03; see Figure 2). Preventing larvae from physically manipulating their environment also reduced sexual size dimorphism in the adult stage (sex‐by‐brood ball modification interaction: *χ* ^2^_(1)_ = 22.31, *p* = <.001, partial *R* ^2^ = .01). This was driven primarily by a stronger reduction in male size in response to the treatments.

### 3.2 Fore tibia length

**FIGURE 3:**
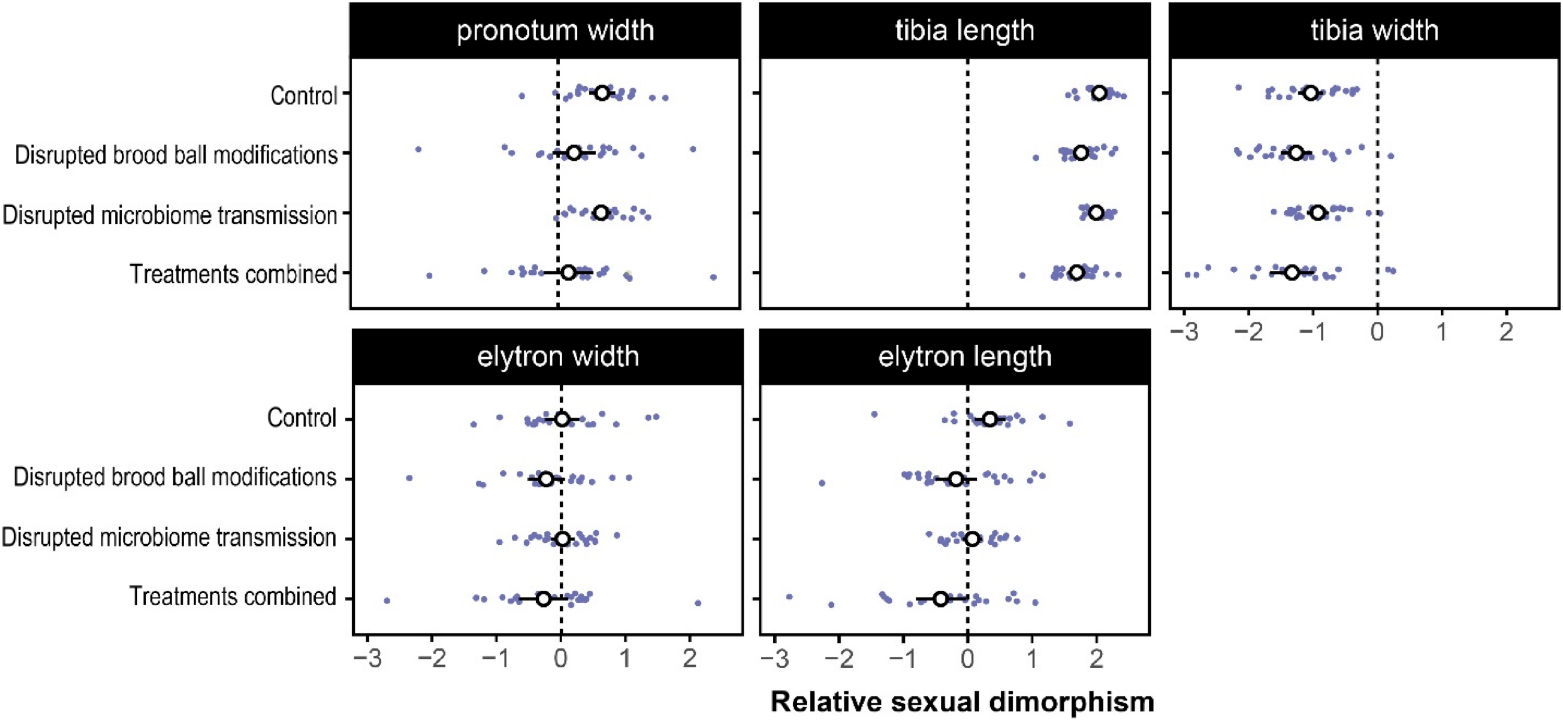
Sexual dimorphism in absolute trait size as a function of the experimental treatment. For each trait, we calculated *z* scores for logarithmized values across sexes and treatments. We then averaged by sire and sex and computed an index for sexual dimorphism by subtracting the mean score for females from that of males (see Perdigon Ferreira et al., 2023 for a similar approach). The resulting index indicated the direction and strength of dimorphism (where values larger than 0 indicate male‐biased sexual dimorphism).

As expected, males had much longer fore tibiae compared to females (*χ* ^2^_(1)_ = 9537.42, *p* = <.001, partial *R* ^2^ = .87; see Figure 3; Figure S2). Absolute tibia length decreased when larvae were limited in their ability to manipulate their brood ball (*χ* ^2^_(1)_ = 149.60, *p* = <.001, partial *R* ^2^ = .02) or when maternal microbiota were withheld (*χ* ^2^_(1)_ = 31.18, *p* = <.001, partial *R* ^2^ = <.01). The response to the disruption of brood ball modifications was stronger in males, leading to decreased sexual dimorphism (*χ* ^2^_(1)_ = 61.31, *p* = <.001, partial *R* ^2^ = <.01). However, fore tibia length shows strong sex‐specific deviations from isometry (allometric slopes in males: 1.46 [1.42, 1.49] 95% confidence limit; females: 0.83 [0.79, 0.87]). When taking body size into account by adding log pronotum width as a covariate (including its interaction with sex), we find that sexual dimorphism in relative tibia length is unaffected by either treatment (see Table S2). These findings contrast to the results of a previous study with a smaller sample size (control and treatment groups combined: 37 males and 33 females) that showed strong sex‐specific effects of developmental niche construction on tibia length in a different population of this species (Schwab et al., 2017). However, a significant main effect of the disruption of brood ball modification on relative tibia length persisted (*χ* ^2^_(1)_ = 51.82, *p* = <.001, partial *R* ^2^ = <.01), indicating that environmental modifications may be involved in the developmental regulation of leg length.

### 3.3 Fore tibia width

In contrast to fore tibia length, sexual dimorphism in relative tibia width was strongly female biased (*χ* ^2^_(1)_ = 1617.68, *p* = <.001, partial *R* ^2^ = .35; Figure 2). Absolute tibia width decreased when larvae were limited in their ability to physically structure their brood ball (*χ* ^2^_(1)_ = 135.15, *p* = <.001, partial *R* ^2^ = .09) and when maternal microbes were removed (*χ* ^2^_(1)_ = 36.07, *p* = <.001, partial *R* ^2^ = .02). The effect of removing brood ball modifications was stronger in males, leading to an increase in sexual dimorphism (*χ* ^2^_(1)_ = 12.32, *p* = <.001, partial *R* ^2^ = <.01). When adding body size as a covariate, the effect of brood ball modifications on sexual dimorphism became non‐significant, although a weak main effect on relative tibia width persisted (*χ* ^2^_(1)_ = 10.01, *p* = .002, partial *R* ^2^ = <.01). Interestingly, we find that sexual dimorphism in relative tibia width was reduced when maternal microbiota were withheld, although this effect was small (*χ* ^2^_(1)_ = 4.46, *p* = .035, partial *R* ^2^ = <.01).

### 3.4 Elytron length

In the control treatment, males had larger elytra compared to females (see Figure 2). Absolute elytron length decreased considerably when brood ball manipulations were disrupted (*χ* ^2^_(1)_ = 89.32, *p* = <.001, partial *R* ^2^ = .12). This effect was stronger in males, leading to a reversal of sexual dimorphism from male biased (in the control treatment) to female biased (*χ* ^2^_(1)_ = 21.53, *p* = <.001, partial *R* ^2^ = .02). Removal of maternal microbiomes also affected elytron length (*χ* ^2^_(1)_ = 40.22, *p* = <.001, partial *R* ^2^ = .02), but this effect did not differ between the sexes (see Table S1). When taking body size into account, these microbiome‐mediated effects on relative elytron length disappeared (Table S2). Females generally had longer elytra relative to body size compared to males in the control treatment, and this sexual dimorphism increased (i.e., became even more female biased) when larvae were prevented from manipulating their brood ball (see Figure S3; sex‐by‐brood ball modification interaction *χ* ^2^_(1)_ = 4.40, *p* = .036, partial *R* ^2^ = <.01).

### 3.5 Elytron width

Females had slightly wider elytra relative to body size compared to males (*χ* ^2^_(1)_ = 127.31, *p* = <.001, partial *R* ^2^ = .05; Figure 3, Figure S2). Absolute elytron width was reduced when microbiota were withheld (*χ* ^2^_(1)_ = 22.44, *p* = <.001, partial *R* ^2^ = .01) and brood ball manipulations were curtailed (*χ* ^2^_(1)_ = 96.24, *p* = <.001, partial *R* ^2^ = .15). The response to the absence of brood ball manipulations was again stronger in males, leading to an increase in sexual dimorphism (*χ* ^2^_(1)_ = 7.36, *p* = .007, partial *R* ^2^ = .01). However, all these effects disappeared when size was accounted for (see Table S2).

### 3.6 Male horn length

**FIGURE 4:**
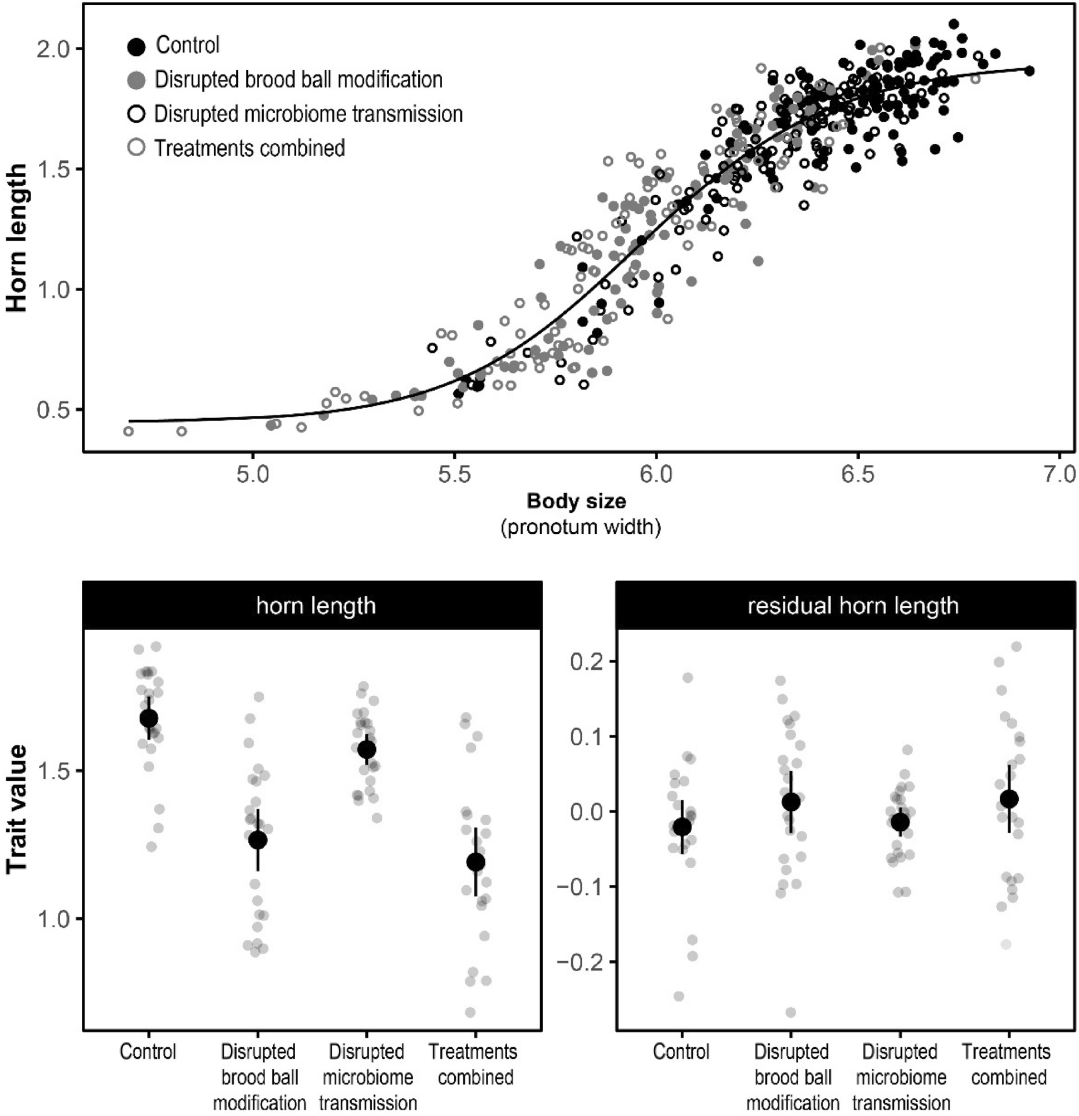
Horn length as a function of experimental treatments (averages and corresponding 95% confidence limits; *n* = 453). The ability of larvae to physically manipulate their brood ball has a large effect on horn length. However, this effect is mediated entirely through changes in overall body size (pronotum width), and relative (or residual) horn size does not change across treatments.

Horn length, a sex‐limited trait, showed a typical sigmoidal scaling relationship with body size (see Figure 4). The different environmental treatments strongly affected absolute horn length (brood ball modification: *χ* ^2^_(1)_ = 121.11, *p* = <.001, partial *R* ^2^ = .23; microbiome treatment: *χ* ^2^_(1)_ = 10.48, *p* = .001, partial *R* ^2^ = .02), but these effects did not persist when accounting for variation due to overall size. We did not find any effects of microbiome or brood ball modification treatments on residual horn length in a mixed model approach (see Table S3). Similarly, the four‐parameter log‐logistic model with one common allometric relationship had a lower AIC (AIC = −475.0) than a model that included separate curves for each treatment combination (AIC = −472.2). Biotic and physical environmental manipulations thus do not have major effects on *relative* horn length in this species.

Taken together, limiting a larva's ability to actively shape its biotic and physical environment had significant effects on sexual dimorphism in absolute trait size. At the same time, we find limited evidence for a general role of developmental niche construction in the regulation of sexual dimorphic morphology beyond mere size effects.

## 4 DISCUSSION

Secondary sexual traits are recognized for their often heightened sensitivity to environmental conditions (but see Bonduriansky, 2007b; Eberhard et al., 2018). In organisms that have the capacity to shape their ontogenetic environment, developmental niche construction may be especially relevant for secondary sexual trait formation. Here, we tested whether traits that vary in the degree and direction of sex‐specific exaggeration are affected—and to what extent—when a larva's ability to construct its ontogenetic environment is curtailed. We find that preventing larvae from physically manipulating their brood ball and disrupting the maternally inherited relationship with microbial communities negatively affects body size. Similar effects were found for the absolute size of secondary sexual traits, which often responded in a sex‐specific manner. This led to an increase or decrease in sexual dimorphism (fore tibia length and width, respectively) and even a reversal from female biased to male biased (elytron length), which suggests that developmental niche construction plays diverse roles in shaping sexual dimorphism. However, when taking overall body size and sex‐specific non‐isometric scaling into account, many of these effects disappeared or became considerably weaker. This was especially true for the relative size of male‐limited head horns, the most strongly exaggerated trait, where all treatment effects were explained by effects on body size. Taken together, while most effects are driven by responses in size, our findings indicate that the reciprocal relationship between developing organisms and their environment shapes sexual dimorphism and functional morphology in adults with potential consequences for behavioral ecology and fitness.

### 4.1 Developmental niche construction shapes the nutritional quality of the ontogenetic environment

Animals that have adapted to grass‐based diets have evolved specialized mechanisms to deal with their recalcitrant and fibrous diets. Cattle, for instance, largely rely on their specialized multi‐chambered guts, the repeated mechanical chewing of their food, and their symbiotic gut microbiome to digest their challenging diet (Mackie, 2002; Xu et al., 2021). Dung beetles relying on cow dung face the additional challenge of feeding on what remains after a very effective ruminant digests its food. As such, it may not be surprising that dung beetles evolved a similarly specialized feeding ecology. The inheritance of microbial symbionts and the physical manipulations made to the brood balls are likely the two most central components. Schwab et al. (2017) showed that the microbial communities in brood balls that were inhabited by a larva are able to break down a much greater diversity of carbon sources, and to a greater degree, than the microbial communities that exist in a brood ball not modified by a larva. This suggests that larval niche construction benefits the physiological capacity of the entire microbiome within the brood ball, which in turn benefits larval growth. Our findings are consistent with this hypothesis, as the experimental reduction of two distinct components of developmental niche construction generally decreased adult size in all traits measured. Furthermore, the stronger effect of the removal of physical brood ball modifications on males corresponds to a stronger dependence on male size on nutritional quantity (Rohner, 2021). Niche construction thus seems to have major effects on the nutritional quality of dung, causing a series of nutritionally plastic responses.

### 4.2 Developmental niche construction impacts sexual dimorphism, functional morphology, and reproductive tactics

Manipulating a developing larva's ability to shape its ontogenetic environment had strong effects on sexual dimorphism and secondary sexual trait exaggeration in the adult. This is best illustrated by the strong reduction in male horn length when developmental niche construction is impeded (see Figure 4). In this and many other dung beetle species, horn length has a bimodal distribution that separates horned “major” males that engage in dyadic fights over breeding opportunities from hornless “minor” males that primarily sneak copulations and invest in post‐copulatory competition. Because horn length and the number of simultaneously competing hornless sneaker males are predictors of success (at least in the related *O. taurus* and *O. acuminatus* (Emlen, 1997a; Moczek & Emlen, 2000)), changes in horn length may alter the social conditions experienced by both males and females and the relative reproductive success associated with each tactic. We found that the proportion of “major” males decreased from 0.92 [0.86, 0.96] (95% binomial confidence limits calculated with the Wilson method in the R package *binom* (Dorai‐Raj, 2022)) in the control treatment to 0.85 [0.77, 0.90] when microbial communities were manipulated, to 0.52 [0.42, 0.62] when brood ball modifications were disrupted, and to 0.49 [0.39, 0.60] when both treatments were applied simultaneously. Because developmental niche construction affects morph frequency, natural populations experiencing such conditions would be expected to be subject to an altered behavioral ecology. Similar effects could be expected for the length of the fore tibia, which is used by a male to hold onto the female during copulation in this species (Rohner et al., 2021), and to drum (as part of male courtship display) on female elytra in others (Beckers et al., 2017; Kotiaho, 2002). Taken together, the resulting population‐wide changes in the absolute size of functional traits are likely to have major effects on performance, fitness, and possibly even the intensity and form of selection individuals experience.

Sexes often differ in their degree of plasticity to environmental conditions (Rohner et al., 2018; Stillwell et al., 2010). Our findings show that this also includes environmental conditions that are constructed by the developing larva. This may indicate that sex‐specific responses to the manipulation of a larva's ability to manipulate its ontogenetic environment may be common. In this context, the role of host–microbiome relationships in shaping sexual dimorphism is particularly interesting. In our experiment, we only manipulated the part of the microbiome that is vertically transmitted from mother to offspring. Although the precise mechanism and degree of fidelity of transmission remain poorly understood, this suggests a role of ecological inheritance in shaping heritable differences in secondary trait expression and sexual dimorphism. Whether this is also the case in natural populations remains to be investigated. However, at least in managed agricultural settings, these effects are expected because, for instance, veterinary antibiotics not only affect the microbiome of cattle but also that of beetles feeding on the dung of treated cows (Hammer et al., 2016). Given our findings, such anthropogenic disruptions of host–microbiome relationships are likely to cause sex‐specific changes in beetle populations with unclear consequences for their reproductive behavior and ecological function.

### 4.3 Developmental niche construction and trait development

Developmental niche construction may impact development through indirect plastic responses to constructed environments or because niche construction behaviors themselves contain or generate regulatory information (Odling‐Smee et al., 2013; Sultan, 2015). In this study, the effects of niche construction on secondary sexual trait expression and sexual dimorphism were overall strong yet nevertheless highly variable across traits. While this demonstrates that ontogenetic environmental manipulations affect sex‐specific morphologies, this does not require environmental manipulations to play a direct regulatory role. Most secondary sexual traits show sex‐specific responses in absolute trait size to plastic changes in body size (Eberhard et al., 2018; Gould, 1966). Effects on sexual dimorphism may thus be driven by indirect size effects. Indeed, when accounting for the effects of sex‐specific scaling, all treatment effects became much weaker and partially disappeared. This was especially the case for male head horns, where the strong treatment effects are entirely explained by effects on body size (Figure 4). This contrasts with the findings of previous studies that demonstrate the dependence of horn allometry on environmental variables. For instance, the horn allometry of *Onthophagus acuminatus* is dependent on dung quality (Emlen, 1997b), and in *Onthophagus taurus*, horn length allometry has been shown to vary in response to the type of larval nutrition and temperature (Moczek, 2002; Rohner & Moczek, 2023). As such, the main effects of developmental niche construction on secondary sexual trait expression seem to be mainly driven by trait‐ and sex specific plastic responses to the constructed nutritional environment.

While most of the niche construction effects documented here can be explained by effects on body size and sex‐specific trait scaling, we also observed several scaling‐independent patterns. This includes significant effects of physical environmental modifications on the relative length and width of the fore tibia and sexual dimorphism in elytron length and tibia width. This suggests that developmental niche construction does have some developmental effects that go beyond mere effects on overall body size plasticity and non‐isometric scaling. However, while significant, most of these effects explained comparably small amounts of variation and were not particularly pronounced in the sex or trait that were more exaggerated. We thus find limited evidence for a strong and general role of ontogenetic environmental modifications in shaping sex‐specific development.

### 4.4 Developmental niche construction and the cost of secondary sexual trait exaggeration

We predicted strongly exaggerated traits to show the strongest responses to the presence or absence of ontogenetic environmental modifications, while we expected little to no effect on weakly sexually dimorphic traits. As predicted, we found very strong effects for the male‐limited head horns (see above), yet effects on other traits were mixed. For instance, changes in the length and width of the weakly dimorphic elytron were similar in magnitude to the changes in the strongly dimorphic fore tibia. The relationship between patterns of sex‐specific selection and responses to the absence of developmental niche construction may thus be more complex than expected.

We based our initial hypothesis on the assumption that individuals adjust trait exaggeration according to environmental quality, which in turn is shaped by developmental niche construction. Such condition‐dependent signaling evolves due to adaptive plasticity that resolves trade‐offs (differential marginal signaling costs for high‐ or low‐quality individuals) or because signals are intrinsically unfakeable (i.e., they underlie inescapable constraints; Penn & Szamado, 2020). If developmental niche construction allows individuals to attribute more resources to an unfakeable signal, it is expected to have major effects on trait expression. The development of horns is developmentally linked to insulin signaling, which in turn is dependent on nutritional status and was therefore suggested to represent a physiologically constrained and intrinsically unfakeable, honest signal (Emlen et al., 2012). However, subsequent work showed that small individuals can indeed develop exaggerated horns, for instance, following functional genetic manipulations of the hedgehog signaling pathway (Kijimoto & Moczek, 2016). These results suggested that rather than not being able to form exaggerated horns, small, low‐nutrition males are actively inhibiting their formation (Rohner et al., 2023). Similarly, the location of the horn threshold is both environmentally plastic (Moczek, 2002; Rohner & Moczek, 2023) and diversifies among populations (Macagno et al., 2021). Horn expression is thus unlikely to be shaped by hard physiological constraints but is more likely driven by differential marginal costs. If so, niche construction's role in trait exaggeration may be more complex. Future work will be necessary to study the role of developmental niche construction in shaping the fitness consequences of secondary sexual trait development.

## 5 CONCLUSIONS

There is a growing appreciation of organisms' abilities to shape the environmental conditions they experience and respond to during ontogeny. Yet, how this capacity impacts phenotypic diversity and functional ecology remains contentious. We here show that developmental niche construction has major sex‐specific effects on phenotypic variation in the size of secondary sexual traits, including quantitative and qualitative changes in sexual dimorphism. However, most of these effects are driven by trait‐ and sex‐specific plastic responses to the constructed nutritional environment, and not a direct involvement of niche construction in the actual developmental regulation of secondary sexual traits. Nevertheless, niche construction‐mediated shifts in the absolute size of functional traits are likely to have important functional ecological and behavioral consequences if they occur in nature. Taken together, our findings suggest that the interactions between developing organisms and their biotic and physical environment can have major impacts on phenotypic variation, even if driven by indirect nutritional effects. Whether this is the case for other taxa or traits remains to be documented.

## AUTHOR CONTRIBUTIONS

**Patrick T. Rohner:** Conceptualization (equal); data curation (equal); formal analysis (equal); funding acquisition (equal); investigation (equal); methodology (equal); project administration (equal); visualization (equal); writing – original draft (equal); writing – review and editing (equal). **Armin P. Moczek:** Conceptualization (equal); project administration (equal); resources (equal); writing – review and editing (equal).

## CONFLICT OF INTEREST STATEMENT

The authors declare no conflicts of interest.

## Supporting information


Data S1

## Data Availability

Raw data are available on Dryad: https://doi.org/10.5061/dryad.pg4f4qrw1.

## References

[ece310666-bib-0001] Bates, D. , Maechler, M. , Bolker, B. , & Walker, S. (2015). Fitting linear mixed‐effects models using lme4. Journal of Statistical Software, 67(1), 1–48.

[ece310666-bib-0002] Beckers, O. M. , Kijimoto, T. , & Moczek, A. P. (2017). Doublesex alters aggressiveness as a function of social context and sex in the polyphenic beetle *Onthophagus taurus* . Animal Behaviour, 132, 261–269. 28966347 10.1016/j.anbehav.2017.08.011 PMC5618252

[ece310666-bib-0003] Bonduriansky, R. (2007a). The evolution of condition‐dependent sexual dimorphism. The American Naturalist, 169, 9–19. 10.1086/510214 17206580

[ece310666-bib-0004] Bonduriansky, R. (2007b). Sexual selection and allometry: A critical reappraisal of the evidence and ideas. Evolution, 61, 838–849. 17439616 10.1111/j.1558-5646.2007.00081.x

[ece310666-bib-0005] Casasa, S. , Zattara, E. E. , & Moczek, A. P. (2020). Nutrition‐responsive gene expression and the developmental evolution of insect polyphenism. Nature Ecology & Evolution, 4, 970–978. 32424280 10.1038/s41559-020-1202-x

[ece310666-bib-0006] Clark, A. D. , Deffner, D. , Laland, K. , Odling‐Smee, J. , & Endler, J. (2020). Niche construction affects the variability and strength of natural selection. The American Naturalist, 195, 16–30. 10.1086/706196 31868536

[ece310666-bib-0007] Cotton, S. , Fowler, K. , & Pomiankowski, A. (2004). Condition dependence of sexual ornament size and variation in the stalk‐eyed fly *Cyrtodiopsis dalmanni* (Diptera: Diopsidae). Evolution, 58, 1038–1046. 15212384 10.1111/j.0014-3820.2004.tb00437.x

[ece310666-bib-0008] Donohue, K. (2005). Niche construction through phenological plasticity: Life history dynamics and ecological consequences. The New Phytologist, 166, 83–92. 15760353 10.1111/j.1469-8137.2005.01357.x

[ece310666-bib-0009] Dorai‐Raj, S. (2022). binom: Binomial confidence intervals for several parameterizations . R Package Version 1.1‐1.1. https://CRAN.R‐project.org/package=binom

[ece310666-bib-0010] Dury, G. J. , Moczek, A. P. , & Schwab, D. B. (2020). Maternal and larval niche construction interact to shape development, survival, and population divergence in the dung beetle *Onthophagus taurus* . Evolution & Development, 22, 358–369. 33448595 10.1111/ede.12348

[ece310666-bib-0011] Eberhard, W. G. , Rodriguez, R. L. , Huber, B. A. , Speck, B. , Miller, H. , Buzatto, B. A. , & Machado, G. (2018). Sexual selection and static allometry: The importance of function. Quarterly Review of Biology, 93, 207–250.

[ece310666-bib-0012] Emlen, D. J. (1997a). Alternative reproductive tactics and male‐dimorphism in the horned beetle *Onthophagus acuminatus* (Coleoptera: Scarabaeidae). Behavioral Ecology and Sociobiology, 41, 335–341.

[ece310666-bib-0013] Emlen, D. J. (1997b). Diet alters male horn allometry in the beetle *Onthophagus acuminatus* (Coleoptera: Scarabaeidae). Proceedings of the Royal Society of London, Series B: Biological Sciences, 264, 567–574.

[ece310666-bib-0014] Emlen, D. J. (1997c). Environmental control of horn length dimorphism in the beetle *Onthophagus acuminatus* (Coleoptera: Scarabaeidae). Proceedings of the Royal Society of London, Series B: Biological Sciences, 256, 131–136.

[ece310666-bib-0015] Emlen, D. J. , Warren, I. A. , Johns, A. , Dworkin, I. , & Lavine, L. C. (2012). A mechanism of extreme growth and reliable signaling in sexually selected ornaments and weapons. Science, 337, 860–864. 22837386 10.1126/science.1224286

[ece310666-bib-0016] Estes, A. M. , Hearn, D. J. , Snell‐Rood, E. C. , Feindler, M. , Feeser, K. , Abebe, T. , Dunning Hotopp, J. C. , & Moczek, A. P. (2013). Brood ball‐mediated transmission of microbiome members in the dung beetle, *Onthophagus taurus* (Coleoptera: Scarabaeidae). PLoS One, 8, e79061. 24223880 10.1371/journal.pone.0079061 PMC3815100

[ece310666-bib-0017] Gilbert, S. F. , Bosch, T. C. , & Ledon‐Rettig, C. (2015). Eco‐Evo‐devo: Developmental symbiosis and developmental plasticity as evolutionary agents. Nature Reviews. Genetics, 16, 611–622. 10.1038/nrg3982 26370902

[ece310666-bib-0018] Gould, S. J. (1966). Allometry and size in ontogeny and phylogeny. Biological Reviews, 41, 587–640. 5342162 10.1111/j.1469-185x.1966.tb01624.x

[ece310666-bib-0019] Grafen, A. (1990). Biological signals as handicaps. Journal of Theoretical Biology, 144, 517–546. 2402153 10.1016/s0022-5193(05)80088-8

[ece310666-bib-0020] Hammer, T. J. , Fierer, N. , Hardwick, B. , Simojoki, A. , Slade, E. , Taponen, J. , Viljanen, H. , & Roslin, T. (2016). Treating cattle with antibiotics affects greenhouse gas emissions, and microbiota in dung and dung beetles. Proceedings of the Royal Society B: Biological Sciences, 283, 20160150. 10.1098/rspb.2016.0150 PMC4892788 27226475

[ece310666-bib-0021] Hanski, I. , & Cambefort, Y. (1991). Dung beetle ecology. Princeton University Press.

[ece310666-bib-0022] Kijimoto, T. , & Moczek, A. P. (2016). Hedgehog signaling enables nutrition‐responsive inhibition of an alternative morph in a polyphenic beetle. Proceedings of the National Academy of Sciences of the United States of America, 113, 5982–5987. 27162357 10.1073/pnas.1601505113 PMC4889385

[ece310666-bib-0023] Kotiaho, J. S. (2002). Sexual selection and condition dependence of courtship display in three species of horned dung beetles. Behavioral Ecology, 13, 791–799.

[ece310666-bib-0024] Kuznetsova, A. , Brockhoff, P. B. , & Christensen, R. H. B. (2017). lmerTest package: Tests in linear mixed effects models. Journal of Statistical Software, 82(13), 1–26.

[ece310666-bib-0025] Linz, D. M. , Hu, Y. , & Moczek, A. P. (2019). The origins of novelty from within the confines of homology: The developmental evolution of the digging tibia of dung beetles. Proceedings of the Royal Society B: Biological Sciences, 286, 20182427. 10.1098/rspb.2018.2427 PMC6408602 30963933

[ece310666-bib-0026] Macagno, A. L. , Moczek, A. P. , & Pizzo, A. (2016). Rapid divergence of nesting depth and digging appendages among tunneling dung beetle populations and species. The American Naturalist, 187, E143–E151. 10.1086/685776 27105002

[ece310666-bib-0027] Macagno, A. L. M. , Edgerton, T. J. , & Moczek, A. P. (2021). Incipient hybrid inferiority between recently introduced, diverging dung beetle populations. Biological Journal of the Linnean Society, 132, 931–944.

[ece310666-bib-0028] Macagno, A. L. M. , & Moczek, A. P. (2022). Between‐partner concordance of vertically transmitted gut microbiota diminishes reproductive output in the dung beetle *Onthophagus taurus* . Physiological Entomology, 48, 14–23.

[ece310666-bib-0029] Mackie, R. I. (2002). Mutualistic fermentative digestion in the gastrointestinal tract: Diversity and evolution. Integrative and Comparative Biology, 42, 319–326. 21708724 10.1093/icb/42.2.319

[ece310666-bib-0030] Moczek, A. P. (1998). Horn polyphenism in the beetle *Onthophagus taurus*: Larval diet quality and plasticity in parental investment determine adult body size and male horn morphology. Behavioral Ecology, 9, 636–641.

[ece310666-bib-0031] Moczek, A. P. (2002). Allometric plasticity in a polyphenic beetle. Ecological Entomology, 27, 58–67.

[ece310666-bib-0032] Moczek, A. P. , & Emlen, D. J. (2000). Male horn dimorphism in the scarab beetle, *Onthophagus taurus*: Do alternative reproductive tactics favour alternative phenotypes? Animal Behaviour, 59, 459–466. 10675268 10.1006/anbe.1999.1342

[ece310666-bib-0033] O'Brien, D. M. , Boisseau, R. P. , Duell, M. , McCullough, E. , Powell, E. C. , Somjee, U. , Solie, S. , Hickey, A. J. , Holwell, G. I. , Painting, C. J. , & Emlen, D. J. (2019). Muscle mass drives cost in sexually selected arthropod weapons. Proceedings of the Royal Society B: Biological Sciences, 286, 20191063. 10.1098/rspb.2019.1063 PMC6599981 31238851

[ece310666-bib-0034] Odling‐Smee, J. , Erwin, D. H. , Palkovacs, E. P. , Feldman, M. W. , & Laland, K. N. (2013). Niche construction theory: A practical guide for ecologists. The Quarterly Review of Biology, 88, 4–28. 23653966 10.1086/669266

[ece310666-bib-0035] Parker, E. S. , Dury, G. J. , & Moczek, A. P. (2019). Transgenerational developmental effects of species‐specific, maternally transmitted microbiota in *Onthophagus* dung beetles. Ecological Entomology, 44, 274–282.

[ece310666-bib-0036] Parker, E. S. , & Moczek, A. P. (2020). Don't stand so close to me: Microbiota‐facilitated enemy release dynamics in introduced *Onthophagus taurus* dung beetles. Ecology and Evolution, 10, 13640–13648. 33391669 10.1002/ece3.6836 PMC7771182

[ece310666-bib-0037] Parker, E. S. , Moczek, A. P. , & Macagno, A. L. M. (2021). Reciprocal microbiome transplants differentially rescue fitness in two syntopic dung beetle sister species (Scarabaeidae: *Onthophagus*). Ecological Entomology, 46, 946–954.

[ece310666-bib-0038] Parker, E. S. , Newton, I. L. G. , & Moczek, A. P. (2020). (my microbiome) would walk 10,000 miles: Maintenance and turnover of microbial communities in introduced dung beetles. Microbial Ecology, 80, 435–446. 32314003 10.1007/s00248-020-01514-9

[ece310666-bib-0039] Penn, D. J. , & Szamado, S. (2020). The handicap principle: How an erroneous hypothesis became a scientific principle. Biological Reviews, 95, 267–290. 31642592 10.1111/brv.12563 PMC7004190

[ece310666-bib-0040] Perdigon Ferreira, J. , Rohner, P. T. , & Lupold, S. (2023). Strongly sexually dimorphic forelegs are not more condition‐dependent than less dimorphic traits in *Drosophila prolongata* . Evolutionary Ecology, 37, 493–508. 37152714 10.1007/s10682-022-10226-0 PMC10156779

[ece310666-bib-0041] Pfennig, D. W. (2021). Phenotypic plasticity & evolution: Causes, consequences, controversies. CRC Press.

[ece310666-bib-0042] Pryke, S. R. , & Andersson, S. (2005). Experimental evidence for female choice and energetic costs of male tail elongation in red‐collared widowbirds. Biological Journal of the Linnean Society, 86, 35–43.

[ece310666-bib-0043] R Core Team . (2021). R: A language and environment for statistical computing. R Foundation for Statistical Computing. https://www.R‐project.org/

[ece310666-bib-0044] Rhebergen, F. T. , Stewart, K. A. , & Smallegange, I. M. (2022). Nutrient‐dependent allometric plasticity in a male‐diphenic mite. Ecology and Evolution, 12, e9145. 35928796 10.1002/ece3.9145 PMC9343935

[ece310666-bib-0045] Ritz, C. , Baty, F. , Streibig, J. C. , & Gerhard, D. (2015). Dose‐response analysis using R. PLoS One, 10, e0146021. 26717316 10.1371/journal.pone.0146021 PMC4696819

[ece310666-bib-0046] Rohlf, F. J. (2009). TpsDig . Department of Ecology and Evolution, State University of New York.

[ece310666-bib-0047] Rohner, P. T. (2021). A role for sex‐determination genes in life history evolution? Doublesex mediates sexual size dimorphism in the gazelle dung beetle. Journal of Evolutionary Biology, 34, 1326–1332. 34075658 10.1111/jeb.13877

[ece310666-bib-0048] Rohner, P. T. , & Blanckenhorn, W. U. (2018). A comparative study of the role of sex‐specific condition dependence in the evolution of sexually dimorphic traits. The American Naturalist, 192, E202–E215. 10.1086/700096 30444660

[ece310666-bib-0068] Rohner, P. T. , Casasa, S. , & Moczek, A. P. (2023). Assessing the evolutionary lability of insulin signaling in the regulation of nutritional plasticity across traits and species of horned dung beetles. Journal of Evolutionary Biology. In press. 10.1111/jeb.14240 37885148

[ece310666-bib-0049] Rohner, P. T. , Linz, D. M. , & Moczek, A. P. (2021). Doublesex mediates species‐, sex‐, environment‐ and trait‐specific exaggeration of size and shape. Proceedings of the Royal Society B: Biological Sciences, 288, 20210241. 10.1098/rspb.2021.0241 PMC8220263 34157867

[ece310666-bib-0050] Rohner, P. T. , Macagno, A. L. M. , & Moczek, A. P. (2020). Evolution and plasticity of morph‐specific integration in the bull‐headed dung beetle *Onthophagus taurus* . Ecology and Evolution, 10, 10558–10570. 33072280 10.1002/ece3.6711 PMC7548182

[ece310666-bib-0051] Rohner, P. T. , & Moczek, A. P. (2020). Rapid differentiation of plasticity in life history and morphology during invasive range expansion and concurrent local adaptation in the horned beetle *Onthophagus taurus* . Evolution, 74, 2059–2072. 32558925 10.1111/evo.14045

[ece310666-bib-0052] Rohner, P. T. , & Moczek, A. P. (2021). Evolutionary and plastic variation in larval growth and digestion reveal the complex underpinnings of size and age at maturation in dung beetles. Ecology and Evolution, 11, 15098–15110. 34765163 10.1002/ece3.8192 PMC8571579

[ece310666-bib-0053] Rohner, P. T. , & Moczek, A. P. (2023). Allometric plasticity and the evolution of environment‐by‐environment (ExE) interactions during a rapid range expansion of a dung beetle. Evolution, 77, 682–689. 36626800 10.1093/evolut/qpac071

[ece310666-bib-0054] Rohner, P. T. , Teder, T. , Esperk, T. , Lupold, S. , & Blanckenhorn, W. U. (2018). The evolution of male‐biased sexual size dimorphism is associated with increased body size plasticity in males. Functional Ecology, 32, 581–591.

[ece310666-bib-0055] Rowe, L. , & Houle, D. (1996). The lek paradox and the capture of genetic variance by condition dependent traits. Proceedings of the Royal Society B: Biological Sciences, 263, 1415–1421.

[ece310666-bib-0056] Ruell, E. W. , Handelsman, C. A. , Hawkins, C. L. , Sofaer, H. R. , Ghalambor, C. K. , & Angeloni, L. (2013). Fear, food and sexual ornamentation: Plasticity of colour development in Trinidadian guppies. Proceedings of the Royal Society B: Biological Sciences, 280, 20122019. 10.1098/rspb.2012.2019 PMC3619452 23466982

[ece310666-bib-0057] Schwab, D. B. , Casasa, S. , & Moczek, A. P. (2017). Evidence of developmental niche construction in dung beetles: Effects on growth, scaling and reproductive success. Ecology Letters, 20, 1353–1363. 28942603 10.1111/ele.12830

[ece310666-bib-0058] Schwab, D. B. , Riggs, H. E. , Newton, I. L. , & Moczek, A. P. (2016). Developmental and ecological benefits of the maternally transmitted microbiota in a dung beetle. The American Naturalist, 188, 679–692. 10.1086/688926 27860508

[ece310666-bib-0059] Shafiei, M. , Moczek, A. P. , & Nijhout, H. F. (2001). Food availability controls the onset of metamorphosis in the dung beetle *Onthophagus taurus* (Coleoptera: Scarabaeidae). Physiological Entomology, 26, 173–180.

[ece310666-bib-0060] Stillwell, R. C. , Blanckenhorn, W. U. , Teder, T. , Davidowitz, G. , & Fox, C. W. (2010). Sex differences in phenotypic plasticity affect variation in sexual size dimorphism in insects: From physiology to evolution. Annual Review of Entomology, 55, 227–245. 10.1146/annurev-ento-112408-085500 PMC4760685 19728836

[ece310666-bib-0061] Stoffel, M. A. , Nakagawa, S. , & Schielzeth, H. (2021). partR2: Partitioning R(2) in generalized linear mixed models. PeerJ, 9, e11414. 34113487 10.7717/peerj.11414 PMC8162244

[ece310666-bib-0062] Stotz, K. (2017). Why developmental niche construction is not selective niche construction: And why it matters. Interface Focus, 7, 20160157. 28839923 10.1098/rsfs.2016.0157 PMC5566811

[ece310666-bib-0063] Sultan, S. E. (2015). Organism and environment: Ecological development, niche construction, and adaption. Oxford University Press.

[ece310666-bib-0064] Uller, T. , & Helantera, H. (2019). Niche construction and conceptual change in evolutionary Biology. British Journal for the Philosophy of Science, 70, 351–375.

[ece310666-bib-0065] West‐Eberhard, M. J. (2003). Developmental plasticity and evolution. OUP.

[ece310666-bib-0066] Xu, Q. , Qiao, Q. , Gao, Y. , Hou, J. , Hu, M. , Du, Y. , Zhao, K. , & Li, X. (2021). Gut microbiota and their role in health and metabolic disease of dairy cow. Frontiers in Nutrition, 8, 701511. 34422882 10.3389/fnut.2021.701511 PMC8371392

[ece310666-bib-0067] Zahavi, A. (1977). The cost of honesty (further remarks on the handicap principle). Journal of Theoretical Biology, 67, 603–605. 904334 10.1016/0022-5193(77)90061-3

