# Supplementary material for: Vertically inherited microbiota and environment‐modifying behaviors indirectly shape the exaggeration of secondary sexual traits in the gazelle dung beetle
Source: Ecol Evol. 2023 Oct 31;13(11):e10666. doi: 10.1002/ece3.10666 (PMC10616735; doi:10.1002/ece3.10666)
Supplement: Supplementary file 1 — Data S1 [file ECE3-13-e10666-s001.docx]

**Supplementary material**

**Vertically inherited microbiota and environment-modifying behaviors indirectly shape the exaggeration of secondary sexual traits in the gazelle dung beetle**

Patrick T. Rohner^1,2^* & Armin Moczek^1^

^1^ Department of Biology, Indiana University Bloomington, Indiana, IN 47405, USA.

^2^ Department of Ecology, Behavior and Evolution, University of California San Diego, La Jolla, CA 92093, USA

*Corresponding author: Patrick T. Rohner, Department of Ecology, Behavior, and Evolution, University of California San Diego, La Jolla, CA 92093, United States; prohner@ucsd.edu.


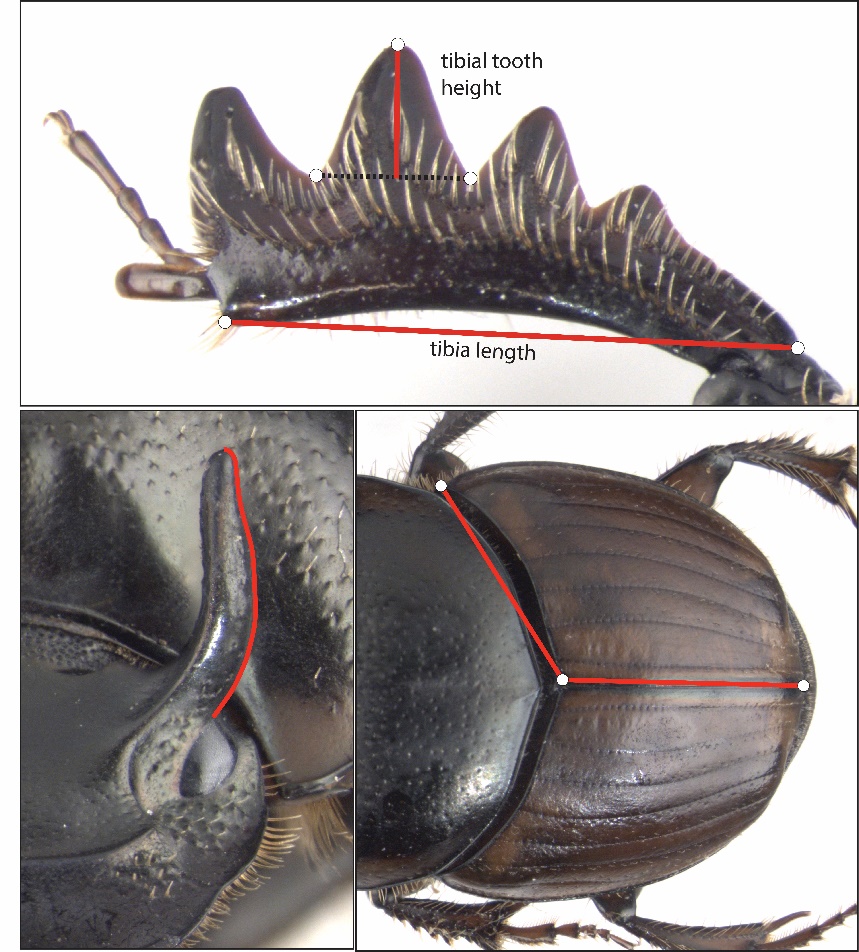


Figure S1: Measurements used in this study. Tibia length was measured as the linear distance between the base and the tip of the foretibia. To calculate tibial tooth height (our estimate for tibia width), we placed one landmarks at the tip of the tooth and two landmarks at the base of the tibia. We then calculated the area of the triangle defined by these three landmarks using Heron’s formula. The height of the tooth was computed by dividing this area by half the distance between the two landmarks on the tibial base. Horn length was estimated as the length of the outline from the eye to the tip of the horn. Elytron length and width was measured as linear distances between landmarks.

**
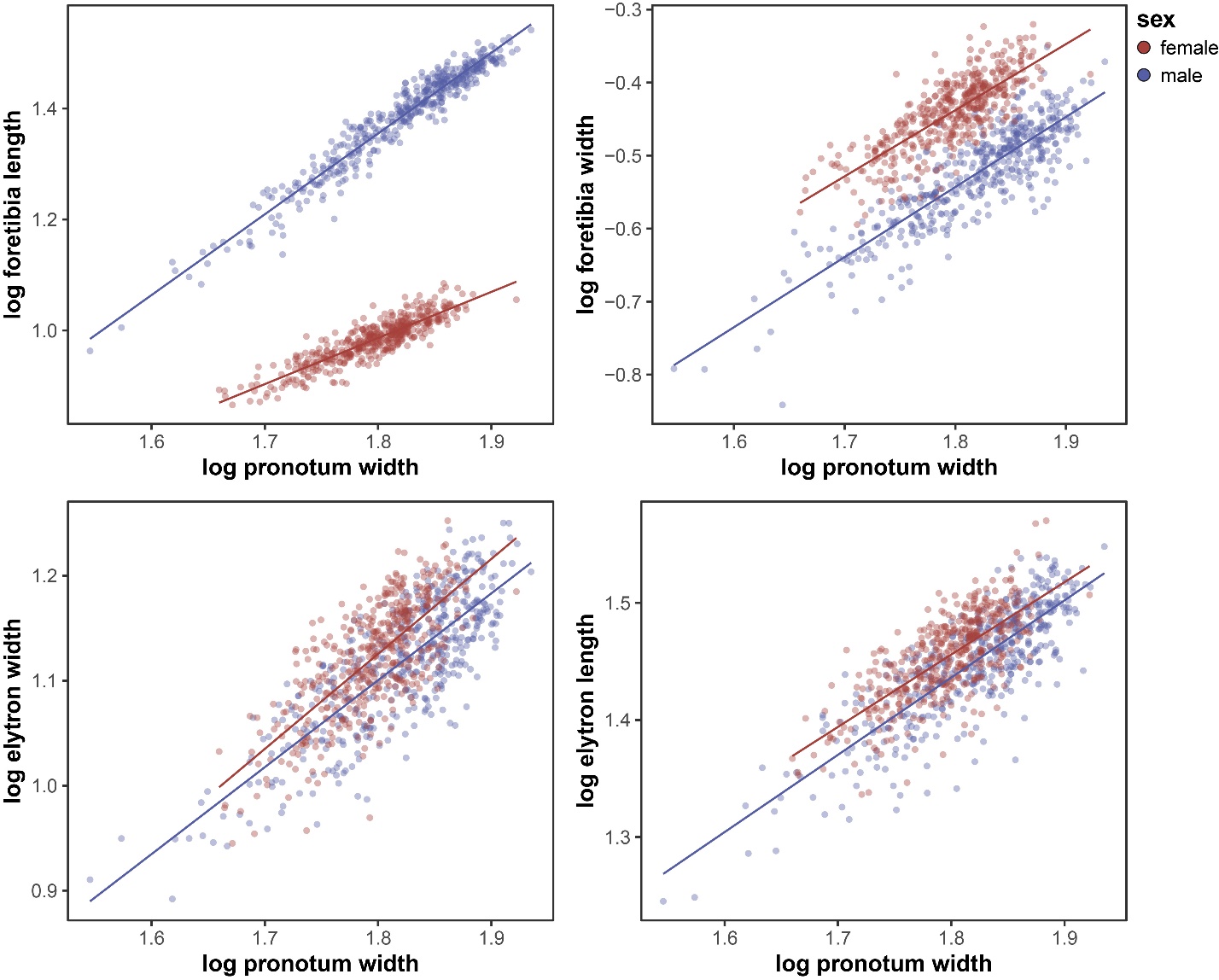
**

Figure S2: Scaling relationship of log trait size with log pronotum width (a suitable estimate of body size in this species, see Rohner et al.(2021)).


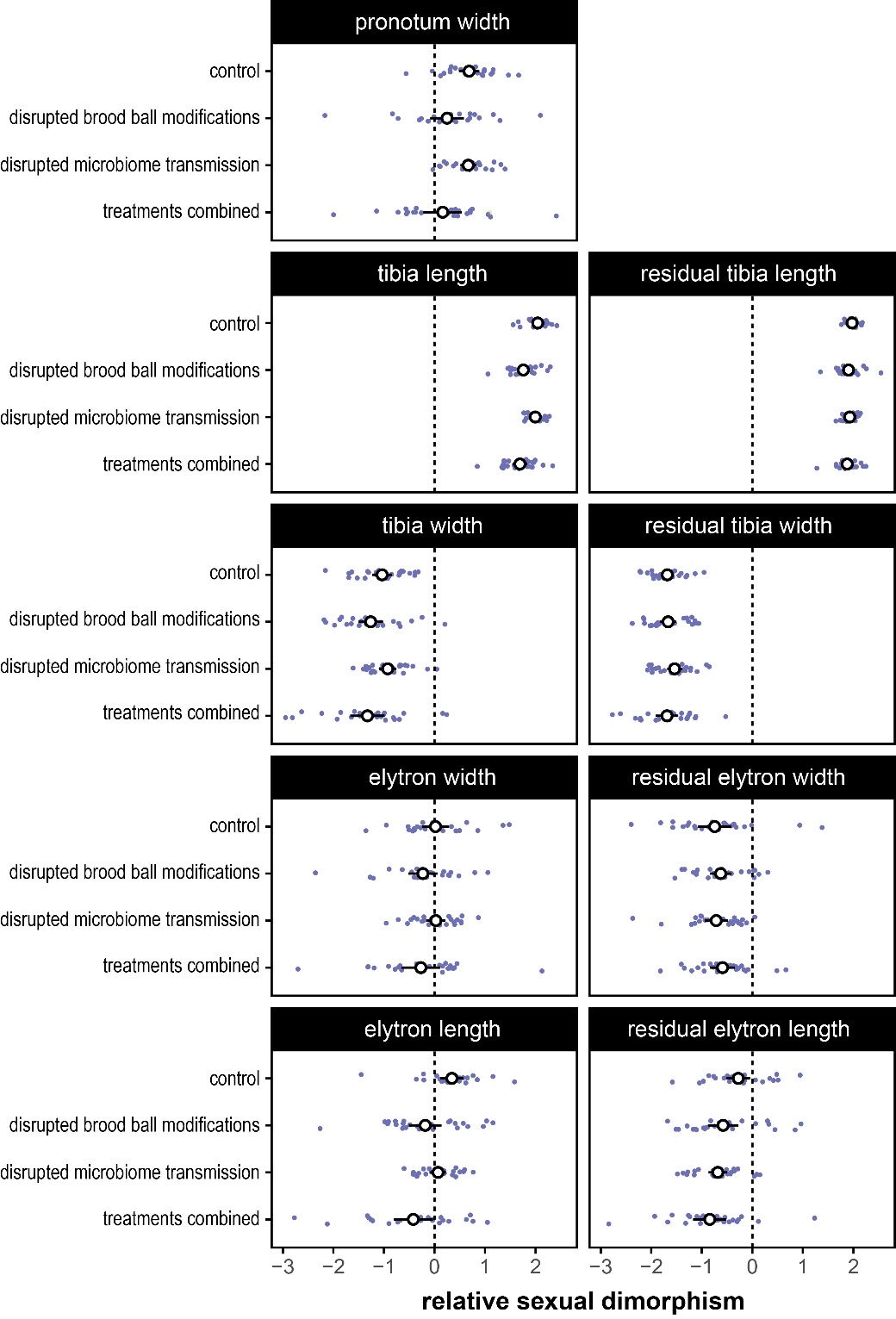


Figure S3: Sexual dimorphism in absolute (left column) and relative (right column) trait size as a function of the experimental treatment combination. For each trait, we calculated z-scores for logarithmized values across sexes and treatments. We then averaged by sire and sex and computed an index for sexual dimorphism by subtracting the mean score for females from that of males. The resulting index indicated the direction and strength of dimorphism (where values larger than 0 indicate male-biased sexual dimorphism).

Table S1: Analysis of Deviance tables (type II Wald chi-square tests) for body size and secondary sexual trait expression as a function of microbiome and brood ball modification treatments. Sire, dam nested within sire, and plate were added as random effect. As approximate effect size for mixed models, we calculated semi-partial R^2^ values following Stoffel et al. (2021) using the *partR2* R package.

| *A) log pronotum width (body size)* | | |  |
| --- | --- | --- | --- |
|  | Chisq | P | part R^2^ |
| sex | 84.42 | <.001 | 0.05 |
| microbiome transmission | 61.64 | <.001 | 0.03 |
| brood ball modification | 246.49 | <.001 | 0.23 |
| sex × microbiome transmission | 0.15 | 0.700 | <.01 |
| sex × brood ball modification | 22.31 | <.001 | 0.01 |
|  |  |  |  |
| *B) log fore tibia length* | | | |
|  | Chisq | P | part R^2^ |
| sex | 9537.42 | <.001 | 0.87 |
| microbiome transmission | 31.18 | <.001 | <.01 |
| brood ball modification | 149.60 | <.001 | 0.02 |
| sex × microbiome transmission | 0.64 | 0.424 | <.01 |
| sex × brood ball modification | 61.31 | <.001 | 0.01 |
| microbiome transmission × brood ball modification | 3.57 | 0.059 | <.01 |
|  |  |  |  |
| *C) log tibial tooth height (fore tibia width)* | | | |
|  | Chisq | P | part R^2^ |
| sex | 623.32 | <.001 | 0.31 |
| microbiome transmission | 36.07 | <.001 | 0.02 |
| brood ball modification | 135.15 | <.001 | 0.09 |
| sex × microbiome transmission | 0.58 | 0.447 | <.01 |
| sex × brood ball modification | 12.32 | <.001 | 0.01 |
|  |  |  |  |
| *D) log elytra length* | | | |
|  | Chisq | P | part R^2^ |
| sex | 1.21 | 0.272 | <.01 |
| microbiome transmission | 40.22 | <.001 | 0.02 |
| brood ball modification | 89.32 | <.001 | 0.12 |
| sex × microbiome transmission | 3.41 | 0.065 | <.01 |
| sex × brood ball modification | 21.53 | <.001 | 0.02 |
|  |  |  |  |
| *E) log elytra width* | | | |
|  | Chisq | P | part R^2^ |
| sex | 2.77 | 0.096 | 0.01 |
| microbiome transmission | 22.44 | <.001 | 0.01 |
| brood ball modification | 96.24 | <.001 | 0.15 |
| sex × microbiome transmission | 0.01 | 0.912 | <.01 |
| sex × brood ball modification | 7.36 | 0.007 | 0.01 |
| microbiome transmission × brood ball modification | 5.16 | 0.023 | <.01 |

Table S2: Analysis of Deviance tables (type II Wald chi-square tests) for relative secondary sexual trait expression as a function of microbiome and brood ball modification treatments. Body size was added as covariate to account for the effect of scaling. Sire, dam nested within sire, and plate were added as random effect. As approximate effect size for mixed models, we calculated semi-partial R^2^ values following Stoffel et al. (2021) using the *partR2* R package.

| *A) log fore tibia length* | | |  |
| --- | --- | --- | --- |
|  | Chisq | P | part R^2^ |
| log pronotum width | 7684.33 | <.001 | 0.08 |
| sex | 82217.57 | <.001 | 0.69 |
| microbiome transmission | 0.14 | 0.712 | <.01 |
| brood ball modification | 51.82 | <.001 | <.01 |
| log pronotum width × sex | 433.46 | <.001 | <.01 |
| sex × microbiome transmission | 2.98 | 0.084 | <.01 |
| sex × brood ball modification | 0.40 | 0.529 | <.01 |
|  |  |  |  |
| *B) log tibial tooth height (fore tibia width)* | | |  |
|  | Chisq | P | part R^2^ |
| log pronotum width | 1617.68 | <.001 | 0.35 |
| sex | 2591.68 | <.001 | 0.50 |
| microbiome transmission | 0.70 | 0.404 | <.01 |
| brood ball modification | 10.01 | 0.002 | <.01 |
| log pronotum width × sex | 5.66 | 0.017 | <.01 |
| sex × microbiome transmission | 4.46 | 0.035 | <.01 |
| sex × brood ball modification | 2.74 | 0.098 | <.01 |
|  |  |  |  |
| *C) log elytra length* | | |  |
|  | Chisq | P | part R^2^ |
| log pronotum width | 1076.84 | <.001 | 0.47 |
| sex | 108.80 | <.001 | 0.05 |
| microbiome transmission | 2.23 | 0.135 | <.01 |
| brood ball modification | 1.84 | 0.175 | <.01 |
| log pronotum width × microbiome transmission | <.01 | 0.965 | <.01 |
| sex × microbiome transmission | 3.19 | 0.074 | <.01 |
| log pronotum width × brood ball modification | 7.92 | 0.005 | <.01 |
| sex × brood ball modification | 4.40 | 0.036 | <.01 |
| microbiome transmission × brood ball modification | 0.59 | 0.441 | <.01 |
| log pronotum width × microbiome transmission × brood ball modification | 3.85 | 0.050 | <.01 |
|  |  |  |  |
| *D) log elytra width* | | |  |
|  | Chisq | P | part R^2^ |
| log pronotum width | 990.00 | <.001 | 0.42 |
| sex | 127.31 | <.001 | 0.05 |
| microbiome transmission | 0.61 | 0.437 | <.01 |
| brood ball modification | 0.12 | 0.724 | <.01 |
| sex × microbiome transmission | 0.60 | 0.440 | <.01 |
| sex × brood ball modification | 0.76 | 0.382 | <.01 |

Table S3: Analysis of Deviance tables (type II Wald chi-square tests) for absolute and relative horn size as a function of microbiome and brood ball modification treatments. Body size was added as covariate to account for the effect of scaling. Sire, dam nested within sire, and plate were added as random effect. As approximate effect size for mixed models, we calculated semi-partial R^2^ values following Stoffel et al. (2021) using the *partR2* R package. Residual horn length (B) was calculated as the residuals from a 4-parameter log-logistic sigmoidal model with a single curve across treatments (i.e., assuming a common allometry).

| *A) horn length* | | |  |
| --- | --- | --- | --- |
|  | Chisq | P | part R^2^ |
| brood ball modification | 121.11 | <.001 | 0.23 |
| microbiome transmission | 10.48 | 0.001 | 0.02 |
|  | | |  |
| *B) residual horn length* | | | |
|  | Chisq | P | part R^2^ |
| brood ball modification | 2.87 | 0.090 | <.01 |
| microbiome transmission | 0.10 | 0.754 | <.01 |
